# Model-Informed Precision Dosing Improves Outcomes in Patients Receiving Vancomycin for Gram-Positive Infections

**DOI:** 10.1093/ofid/ofae002

**Published:** 2024-01-05

**Authors:** Nicole M Hall, Matthew L Brown, W Seth Edwards, Robert A Oster, Will Cordell, Joshua Stripling

**Affiliations:** Department of Pharmacy, UAB Hospital, Birmingham, Alabama, USA; Department of Pharmacy, UAB Hospital, Birmingham, Alabama, USA; Department of Pharmacy, UAB Hospital, Birmingham, Alabama, USA; Department of Medicine, Division of Preventive Medicine, University of Alabama at Birmingham, Birmingham, Alabama, USA; Department of Pharmacy, The University of Kansas Health System, Kansas City, Kansas, USA; Division of Infectious Diseases, Department of Medicine, University of Alabama at Birmingham Heersink School of Medicine, Birmingham, Alabama, USA

**Keywords:** area under the curve, AUC, model-informed precision dosing, therapeutic drug monitoring, vancomycin

## Abstract

**Background:**

Consensus guidelines for dosing and monitoring of vancomycin recommend collection of 2 serum concentrations to estimate an area under the curve/minimum inhibitory concentration ratio (AUC/MIC). Use of Bayesian software for AUC estimation and model-informed precision dosing (MIPD) enables pre–steady state therapeutic drug monitoring using a single serum concentration; however, data supporting this approach are limited.

**Methods:**

Adult patients with culture-proven gram-positive infections treated with vancomycin ≥72 hours receiving either trough-guided or AUC-guided therapy were included in this retrospective study. AUC-guided therapy was provided using MIPD and single-concentration monitoring. Treatment success, vancomycin-associated acute kidney injury (VA-AKI), and inpatient mortality were compared using a desirability of outcome ranking analysis. The most desirable outcome was survival with treatment success and no VA-AKI, and the least desirable outcome was death.

**Results:**

The study population (N = 300) was comprised of an equal number of patients receiving AUC-guided or trough-guided therapy. More patients experienced the most desirable outcome in the AUC-guided group compared to the trough-guided group (58.7% vs 46.7%, *P* = .037). Rates of VA-AKI were lower (21.3% vs 32.0%, *P* = .037) and median hospital length of stay was shorter (10 days [interquartile range {IQR}, 8–20] vs 12 days [IQR, 8–25]; *P* = .025) among patients receiving AUC-guided therapy.

**Conclusions:**

AUC-guided vancomycin therapy using MIPD and single-concentration monitoring improved outcomes in patients with culture-proven gram-positive infections. Safety was improved with reduced incidence of VA-AKI, and no concerns for reduced efficacy were observed. Moreover, MIPD allowed for earlier assessment of AUC target attainment and greater flexibility in the collection of serum vancomycin concentrations.

## Teaser

Area under the curve (AUC)–guided vancomycin therapy via model-informed precision dosing using pre–steady state, single-concentration monitoring for culture-proven gram-positive infections was compared to those receiving trough-guided therapy using a desirability of outcome ranking analysis. More patients experienced the most desirable outcome with AUC-guided therapy.

## Graphical Abstract

**Graphical Abstract:**
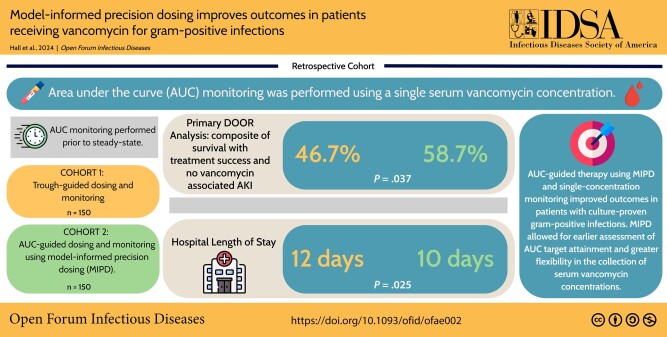
This graphical abstract is also available at Tidbit: https://tidbitapp.io/tidbits/model-informed-precision-dosing-improves-outcomes-in-patients-receiving-vancomycin-for-gram-positive-infections

Vancomycin is the one of the most commonly utilized antibiotics in the hospital setting [1, 2]. Often used empirically for a wide range of infections, it is the drug of choice for severe methicillin-resistant *Staphylococcus aureus* infections [3]. However, vancomycin use is not without risk, most notably nephrotoxicity [4]. Previously reported incidence of vancomycin-associated acute kidney injury (VA-AKI) ranges between 5% and 43% depending on additional risk factors and pharmacokinetic drug exposure [5–7]. Trough concentrations ≥15 μg/mL and area under the curve over minimum inhibitory concentration ratios (AUC/MIC) as low as 515 mg/L × hour have been associated with an increased risk of VA-AKI [6, 7]. AUC/MIC is the pharmacokinetic parameter best correlated with the antibacterial activity of vancomycin, and AUC-guided dosing and monitoring has been shown to reduce risk of VA-AKI and improve patient outcomes [8–10]. As a result, consensus guidelines on the dosing and monitoring of vancomycin were updated in 2020 to recommend the use of AUC-guided dosing and monitoring over a trough-guided approach [11].

In addition, a Bayesian approach using 2 pharmacokinetic samples is the preferred method for AUC-guided vancomycin dosing [11]. Bayesian software allows for model-informed precision dosing (MIPD) using a validated pharmacokinetic model in combination with patient-specific characteristics to design dosing regimens intended to achieve the recommended AUC/MIC target of 400–600 mg/L × hour [12]. MIPD is advantageous compared to traditional approaches to vancomycin dosing, including the use of trough concentrations or first-order pharmacokinetic equations, in that it allows for greater flexibility of dosing with therapeutic drug monitoring (TDM). Specifically, single serum concentrations may be obtained pre–steady state at any time point following vancomycin administration and pharmacokinetic distribution (ie, during the pharmacokinetic elimination phase which occurs 1–2 hours after the end of the infusion until the next dose is administered). These advantages can allow for early dose optimization, improved logistical workflow, and a simplified approach to implement current consensus guideline recommendations for vancomycin dosing and monitoring.

Currently there is a paucity of clinical data to support the use of single pre–steady state concentrations or single nontrough concentrations to facilitate AUC-guided vancomycin therapy. Neely et al initially highlighted the utility of AUC estimation using a single vancomycin concentration in a cohort of 47 patients by demonstrating that trough-only data produced accurate AUC estimations [13]. Unfortunately, there is a lack of special populations included in this study. More recent data on single-concentration Bayesian AUC estimation are based on a cohort of 63 patients that includes obese and critically ill patients [14]. The results add support for the use of single-concentration (ie, trough-only) monitoring to achieve desired AUC targets; however, clinical outcomes related to this approach were not reported. The purpose of this study is to evaluate the safety and efficacy of AUC-guided vancomycin therapy using MIPD and single-concentration monitoring compared to trough-guided dosing and monitoring in a diverse patient population with culture-proven gram-positive infections.

## METHODS

### Study Design and Population

This was a retrospective, single-center, quasi-experimental study of adult patients with culture-proven gram-positive infections at a large academic medical center between January 2021 and July 2022. Patients were included if they were ≥18 years of age, had a laboratory-confirmed gram-positive infection from any source, and received vancomycin for at least 72 hours. The clinical diagnosis and source of infection were determined by the treating clinician. Patients on renal replacement therapy or with an estimated glomerular filtration rate (eGFR) <30 mL/minute at the time of vancomycin initiation, those with mixed gram-positive and gram-negative infections, and those receiving vancomycin prior to admission were excluded. Cultures considered by the treating clinician to represent contamination were also excluded. Patients in the trough-guided group received dosing regimens based on an institution-specific nomogram, targeting steady-state trough concentrations of 10–20 μg/mL. Patients in the AUC-guided group received dosing regimens based on InsightRX Nova, an electronic health record–integrated Bayesian MIPD software program designed to target an AUC of 400–600 μg/L × hour [15]. Vancomycin pharmacokinetics were based on the Carreno model for patients with a body mass index (BMI) >40 kg/m^2^ or the Goti model without serum creatinine rounding for all other patients [16–18]. Patients in both groups received an initial weight-based loading dose if they met specific criteria according to an institution-specific protocol (systolic blood pressure <90 mm Hg, mean arterial pressure <60 mm Hg, or receipt of vasopressor support at time of vancomycin initiation). Automatic infectious diseases consultation is employed at our institution for patients with *S aureus* or enterococcal bacteremia.

### Outcomes

The primary outcome was a desirability of outcome ranking (DOOR) analysis comparing overall outcomes between groups based on occurrences of treatment success (defined as white blood cell [WBC] count <12 × 10^3^ cells/μL and temperature <38.0°C (<100.4°F) at end of therapy, no switch in therapy due to inadequate response to therapy or toxicity, and no infection- or vancomycin-related readmission by 30 days), vancomycin-associated acute kidney injury (VA-AKI; defined as an increase in serum creatinine by ≥0.3 mg/dL within 48 hours or >1.5× baseline with an onset of >48 hours after vancomycin initiation and up to 48 hours after vancomycin discontinuation), and inpatient mortality. We defined VA-AKI as the onset of an AKI >48 hours after vancomycin initiation based on studies that suggest it to be unlikely for <48 hours of vancomycin therapy to cause an AKI [19]. Baseline serum creatinine was calculated as the median of all serum creatinine measurements prior to the administration of vancomycin, if available, or as the median of all serum creatinine measurements up to 48 hours after vancomycin initiation. Increases in serum creatinine were determined by comparing rises in serum creatinine to any value obtained within the previous 48 hours. Treatment failure was defined as WBC count ≥12 × 10^3^ cells/μL or temperature ≥38.0°C (≥100.4°F) at end of therapy, switch in therapy due to inadequate response to therapy or antibiotic toxicity, or an infection- or vancomycin-related readmission by 30 days. The 5 outcome categories from most to least desirable were (1) survival with treatment success and no VA-AKI; (2) survival with treatment success and VA-AKI; (3) survival with treatment failure and no VA-AKI; (4) survival with treatment failure and VA-AKI; and (5) death. Secondary outcomes included the individual components of the primary outcome, rates of escalation in level of care during the treatment course, 30-day readmission, intensive care unit (ICU) length of stay (LOS), and hospital LOS. To allow for direct comparisons related to AUC attainment between groups, covariates for patients in the trough-guided group were manually input into the InsightRX Nova software program. The study was approved by the Institutional Review Board at the University of Alabama at Birmingham.

### Statistical Analysis

Descriptive statistics were used to summarize categorical variables as frequencies and percentages, and continuous variables as means and standard deviations. Proportions for categorical variables were compared between groups using the χ^2^ test, or Fisher exact test if the assumptions for the χ^2^ test were not satisfied. Means of continuous variables were compared between groups using the 2-group *t* test. Distributions of continuous variables were examined using graphical techniques and tests of normality. Since distributions of a few of the variables deviated from a normal distribution, we concurrently used the Wilcoxon rank-sum test to compare means between groups; as results obtained from the 2-group *t* test and the Wilcoxon rank-sum test were similar, we present the results obtained from the 2-group *t* test for ease of interpretation and comparison with previous literature. We also performed a subanalysis to determine whether there was an association between VA-AKI and either hospital or ICU LOS. Since the LOS variables have highly skewed distributions, we performed analysis of covariance (ANCOVA) using the log-transformed values for the LOS variables and including terms for group (trough-guided, AUC-guided), VA-AKI (yes, no), and a group by VA-AKI interaction in the hospital and ICU LOS ANCOVA models. The point estimate and confidence interval for the better DOOR probability were determined according to Ong et al [20]. No tiebreakers or partial credit scores were used as part of the DOOR analysis. All tests of significance were 2-tailed and a *P* value of <.05 was considered statistically significant. Statistical analyses were performed using SAS software (SAS Institute, Cary, North Carolina), version 9.4.

## RESULTS

**Figure 1.**
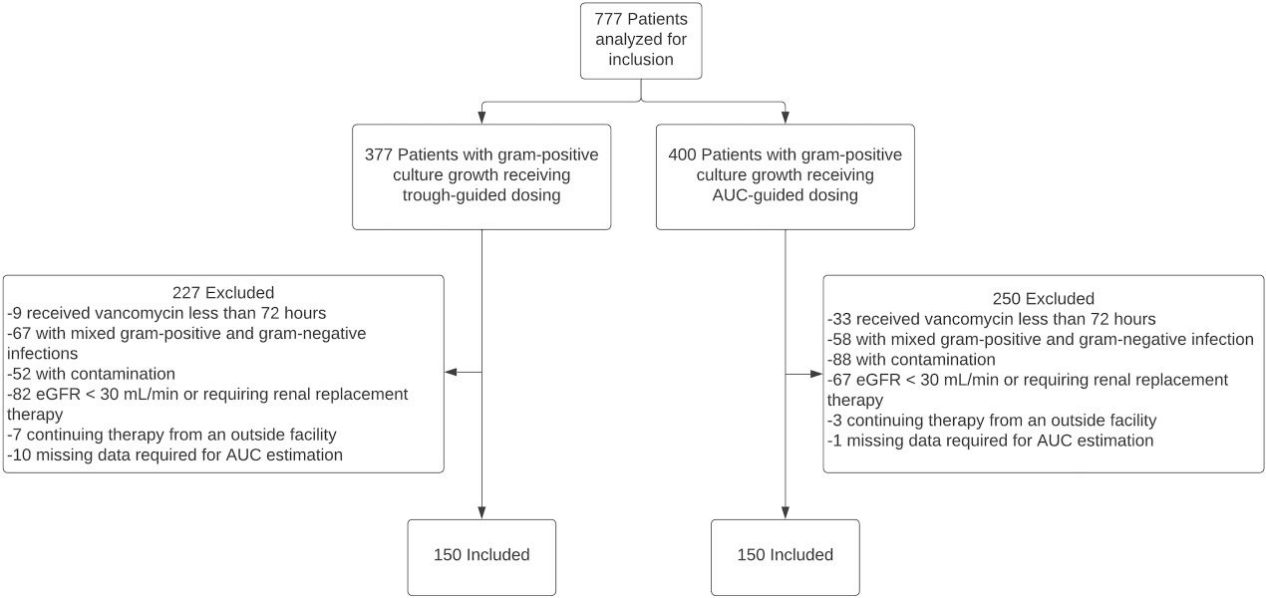
Study population. Abbreviations: AUC, area under the curve, eGFR, estimated glomerular filtration rate.

A total of 777 patients receiving vancomycin for a culture-proven gram-positive infection were evaluated for inclusion in the analysis. After exclusion, a total of 300 patients were included, with an equal number of patients receiving trough-guided or AUC-guided dosing and monitoring (Figure 1). Patient demographics and vancomycin treatment characteristics for the 2 groups are outlined in Table 1. Overall, the groups’ treatment characteristics were well matched. Patients in both groups were predominately male, White, and around 50 years of age. Approximately one-third of patients in each group had a BMI >30 kg/m^2^. More patients in the AUC-guided group had a baseline eGFR of 50–60 mL/minute compared to those in the trough-guided group. All other baseline characteristics, including the percentage of patients requiring vasopressor support, mechanical ventilation, and admission to an ICU at vancomycin initiation, were similar between both groups. The number of patients concomitantly receiving other nephrotoxic agents was high but similar between groups (76.7% vs 70.7% of patients in the trough-guided and AUC-guided groups, respectively).

**Table 1.** Baseline Demographics and Vancomycin Treatment Characteristics

Characteristic	Trough-Guided (n = 150)	AUC-Guided (n = 150)	*P* Value
Age, y, mean ± SD	53 ± 16	55 ± 15	.400
Race			
White	88 (58.7)	95 (63.3)	.407
African American	53 (35.3)	48 (32.0)	.541
Hispanic	4 (2.7)	2 (1.3)	.684
Other	5 (3.3)	5 (3.3)	1.0
Male sex	99 (66.0)	88 (58.7)	.190
Weight, kg, mean ± SD	88.1 ± 25.8	86.2 ± 28.7	.544
BMI, kg/m^2^, mean ± SD	28.3 ± 7.5	28.5 ± 9.1	.852
Baseline eGFR, mL/min			
>60	120 (80.0)	111 (74.0)	.217
50–60	11 (7.3)	24 (16.0)	.019
40–49	6 (4.0)	8 (5.3)	.584
30–39	13 (8.7)	7 (4.7)	.165
Chronic kidney disease	5 (3.3)	13 (8.7)	.052
Diabetes mellitus	47 (31.3)	40 (26.7)	.373
Cardiovascular disease	56 (37.3)	58 (38.7)	.812
Cirrhosis	5 (3.3)	7 (4.7)	.556
IV drug use	11 (7.3)	14 (9.3)	.531
Immunocompromised	22 (14.7)	21 (14.0)	.869
Pitt bacteremia score, mean ± SD	0.59 ± 0.86	0.67 ± 0.97	.451
ICU level of care at vancomycin initiation	53 (35.3)	42 (28.0)	.172
Mechanical ventilation at treatment initiation	24 (16.0)	18 (12.0)	.318
Vasopressor support at treatment initiation	9 (6.0)	9 (6.0)	1.0
Concurrent antimicrobials at treatment initiation	139 (92.7)	133 (88.7)	.234
Infectious diseases consult	117 (78.0)	110 (73.3)	.346
Concurrent nephrotoxins^a^	115 (76.7)	106 (70.7)	.238
Bacteremia	98 (65.3)	97 (64.7)	.904
Source control indicated but not achieved	14 (9.3)	14 (9.3)	1.0
Clinical diagnosis			.131
Endocarditis	4 (2.7)	12 (8.0)	–
Osteomyelitis or prosthetic joint infection	31 (20.7)	19 (12.7)	–
Skin and soft tissue	25 (16.7)	26 (17.3)	–
Line-associated infection	13 (8.7)	8 (5.3)	–
Unknown	33 (22.0)	36 (24.0)	–
Other^b^	44 (29.3)	49 (32.7)	–
Organism			.170
*Staphylococcus aureus*	66 (44.0)	62 (41.3)	–
Methicillin susceptible	15 (10.0)	9 (6.0)	
Methicillin resistant	51 (34.0)	53 (35.3)	
Coagulase-negative staphylococci	41 (27.3)	52 (34.7)	–
*Enterococcus faecalis*	7 (4.7)	6 (4.0)	–
Streptococcus spp	5 (3.3)	11 (7.3)	–
Polymicrobial including *S aureus*	11 (7.3)	10 (6.7)	–
Polymicrobial not including *S aureus*	17 (11.3)	6 (4)	–
Other	3 (2.0)	3 (2.0)	–
Inpatient vancomycin treatment duration, d, mean ± SD	9.6 ± 8.0	8.5 ± 5.6	.164
Receipt of loading dose	13 (8.7)	14 (9.3)	.840
Loading dose, mg/kg, mean ± SD	24.6 ± 2.8	25.3 ± 2.7	.525
Total daily dose of initial regimen, mg/kg, mean ± SD	26.6 ± 11.0	21.7 ± 7.8	<.001
Vancomycin concentrations obtained per course, mean ± SD	3.6 ± 2.6	4.5 ± 2.4	.003
Time to initial vancomycin concentration, h, mean ± SD	44.8 ± 22.3	23.8 ± 14.1	<.001
Obtained within 72 h of first dose, mean ± SD	1.4 ± 0.8	1.9 ± 0.8	<.001
Added to another blood draw, mean ± SD	0.7 ± 0.9	1.7 ± 1.7	<.001

Data are presented as No. (%) unless otherwise indicated.

Abbreviations: AUC, area under the curve; BMI, body mass index; eGFR, estimated glomerular filtration rate; ICU, intensive care unit; IV, intravenous; SD, standard deviation.

^a^Nephrotoxic agents: aminoglycosides, amphotericin B, angiotensin-converting enzyme inhibitors, angiotensin receptor blockers, cidofovir, cisplatin, contrast dye, IV cyclosporine, ganciclovir, interferon-α, lithium, loop diuretics, methotrexate, nonsteroidal anti-inflammatory agents, pentamidine, piperacillin-tazobactam, phenytoin, tenofovir disoproxil fumarate, thiazide diuretics, trimethoprim-sulfamethoxazole, tacrolimus.

^b^Other infection types included pneumonia (trough-guided: n = 7; AUC-guided: n = 10), septic arthritis (trough-guided: n = 5; AUC-guided: n = 4), meningitis (trough-guided: n = 1; AUC-guided: n = 0), urinary tract infections (trough-guided: n = 3; AUC-guided: n = 6), intra-abdominal infections (trough-guided: n = 7; AUC-guided: n = 3), and surgical site infections (trough-guided: n = 10; AUC-guided: n = 8).

The most common pathogen was *S aureus*, but infections with non–*S aureus* isolates made up over half of the cohort. The most common types of infection included osteomyelitis, prosthetic joint infections, and skin and soft tissue infections. Over half of the patients in each group had bacteremia. The majority of patients (78.0% vs 73.3% in the trough-guided and AUC-guided groups, respectively) had an infectious diseases team consultation and achieved source control if indicated (90.7% in both treatment groups).

Given the specific criteria for loading doses outlined in our institutional vancomycin dosing and monitoring protocol, only 13 (8.7%) patients in the trough-guided group and 14 (9.3%) patients in the AUC-guided group received a loading dose. Weight-based loading doses administered were similar between groups on average (25 mg/kg). Average maintenance doses were significantly lower in the AUC-guided group (21.7 mg/kg/day vs 26.6 mg/kg/day, *P* < .001). Patients in the AUC-guided group had TDM performed earlier than patients in the trough-guided group (23.8 hours vs 44.8 hours, respectively; *P* < .001). Use of MIPD in the AUC-guided group allowed for 37.4% of TDM to be performed as an addition to a blood draw originally ordered for other laboratory monitoring compared to only 19.8% in the trough-guided group.

**Figure 2.**
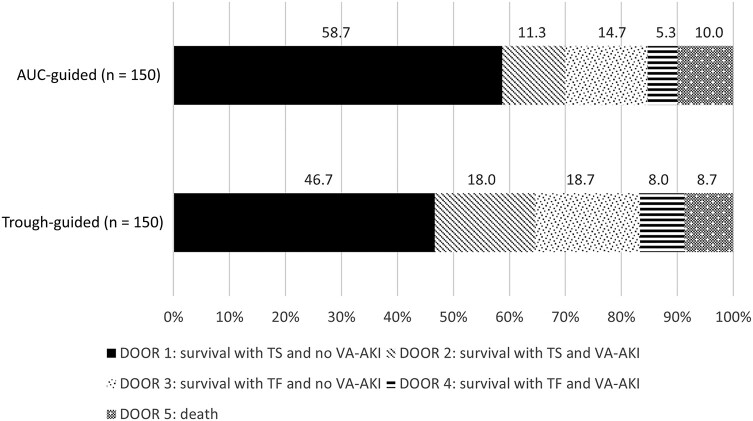
Desirability of outcome ranking analysis. Abbreviations: AUC, area under the curve; DOOR, desirability of outcome ranking; TF, treatment failure; TS, treatment success; VA-AKI, vancomycin-associated acute kidney injury.

The probability that a patient receiving AUC-guided therapy would experience a more desirable outcome than a patient receiving trough-guided therapy was 54.9% (95% confidence interval, 48.9%–60.9%). The distribution of DOOR rankings between the 2 groups is illustrated in Figure 2. More patients in the AUC-guided group experienced the most desirable outcome compared to patients in the trough-guided group (58.7% vs 46.7%, respectively; *P* = .037). Secondary outcomes are displayed in Table 2. Improved outcomes were driven by a significant reduction in VA-AKI in the AUC-guided group compared to the trough-guided group (21.3% vs 32.0%, respectively; *P* = .037). Fewer patients in the AUC-guided group experienced potentially toxic exposures to vancomycin evidenced by a lower rate of trough concentrations ≥15 μg/mL (33.3% vs 51.3%, *P* = .002) and AUC >600 mg/L × hour (22.7% vs 36.7%, *P* = .008). There was a significant reduction in both hospital (10 vs 12 days, *P* = .025) and ICU LOS (8 vs 14 days, *P* = .026) in the AUC-guided group compared to the trough-guided group, respectively. The reduction in hospital LOS observed in the AUC-guided group was significantly associated with reduction in the incidence of VA-AKI according to the ANCOVA model of the subanalysis. The reduction in ICU LOS observed in the AUC-guided group was not clearly associated with reduced rates of VA-AKI.

**Table 2.** Secondary Outcomes

Outcome	Trough-Guided (n = 150)	AUC-Guided (n = 150)	*P* Value
VA-AKI	48 (32.0)	32 (21.3)	.037
AKI stage at peak AKI			
Stage 1	35 (72.9)	25 (78.1)	.598
Stage 2	7 (14.6)	1 (3.1)	.094
Stage 3	6 (12.5)	6 (18.8)	.443
AKI requiring renal replacement therapy	3 (6.3)	2 (6.3)	1.0
Trough during treatment course			
≥15 μg/mL	77 (51.3)	50 (33.3)	.002
15–20 μg/mL	48 (32.0)	36 (24.0)	.123
>20 μg/mL	29 (19.3)	14 (9.3)	.014
AUC >600 mg/L × h during treatment course	55 (36.7)	34 (22.7)	.008
Switch to alternative therapy due to inadequate response to therapy or toxicity	12 (8.0)	10 (6.7)	.658
Duration of bacteremia, d, mean ± SD^a^	4.0 ± 2.6	3.5 ± 2.9	.260
Escalation in level of care during treatment course	7 (4.7)	9 (6.0)	.607
Readmission at 30 d in patients alive at discharge	23/137 (16.8)	22/134 (16.4)	.935
Infection related	7 (5.1)	3 (2.2)	.334
Vancomycin treatment related	0 (0)	0 (0)	NA
Treatment success	102 (68.0)	107 (71.3)	.530
Hospital LOS, d			.025
Mean ± SD	22 ± 34	15 ± 14	
Median (IQR)	12 (8–25)	10 (8–20)	
ICU LOS, d			.026
Mean ± SD^b^	24 ± 43	11 ± 9	
Median (IQR)^b^	14 (6–28)	8 (5–14)	

Data are presented as No. (%) unless otherwise indicated.

Abbreviations: AKI, acute kidney injury; AUC, area under the curve; IQR, interquartile range; LOS, length of stay; NA, not applicable; SD, standard deviation; VA-AKI, vancomycin-associated acute kidney injury.

^a^In the trough-guided group (n = 98) and in the AUC-guided group (n = 97).

^b^In the trough-guided group (n = 65) and in the AUC-guided group (n = 54).

## DISCUSSION

The updated consensus guidelines for vancomycin dosing and monitoring prefer Bayesian-assisted AUC-guided therapy, preferably based on the collection of 2 serum concentrations due to limited data supporting the use of single-concentration monitoring. We demonstrate overall improved patient outcomes with AUC-guided therapy through the use of Bayesian MIPD using single-concentration monitoring compared with trough-guided therapy. Furthermore, our study population was comprised of several patient groups where the benefit of MIPD and single-concentration Bayesian AUC estimation is less established (such as patients who are critically ill or obese). Our study also included patients with non–*S aureus* infections as well as various types of infections (eg, skin and soft tissue infections and bone and joint infections) where overall data for AUC-guided vancomycin therapy are limited.

Furthermore, our study highlights multiple clinical and operational benefits of MIPD using single-concentration monitoring. First, it allowed TDM to be performed approximately 1 day earlier, on average, resulting in earlier dose optimization. We believe this enabled prompt dosage adjustments to balance therapeutic efficacy with risk of toxicity. Second, Bayesian MIPD allows for vancomycin concentrations to be obtained at nearly any time point following administration and pharmacokinetic distribution. This enables orders for vancomycin concentrations to be obtained at times most convenient for nursing, phlebotomy staff, and patients, such as during routine blood draws for other laboratory testing. In addition, single-concentration monitoring offers additional convenience over 2-concentration monitoring as it avoids need for extra blood draws and laboratory testing that could disrupt healthcare provider workflow, decrease patient satisfaction, and increase costs.

Our study has several limitations, most notably the retrospective and single-center study design. A high, but similar, percentage of patients in each group received concurrent antimicrobial therapy at the time of vancomycin initiation. We believe this represents real-world clinical practice related to the use of broad-spectrum empiric antibiotic therapy; however, the study was not designed to evaluate the impact of concurrent antibiotic therapy on the outcomes assessed. Furthermore, the heterogeneity of clinical diagnoses included made it difficult to definitively assess treatment success or failure for some types of infection. This study was also not designed to evaluate the performance of specific pharmacokinetic models in the attainment of the target AUC range or the impact of specific models on patient outcomes. Such future evaluations may assist with further optimization of MIPD of vancomycin. Additional studies comparing clinical, operational, and economic outcomes between MIPD single- and 2-concentration monitoring may better inform the preferred approach to AUC-guided vancomycin therapy.

Following release of the updated vancomycin consensus guideline recommendations, questions regarding the true impact on patient outcomes of AUC-guided dosing over trough-guided dosing were posed [21]. Our study demonstrates an improvement in overall favorable clinical outcomes and a significant reduction in both hospital and ICU LOS with the use of AUC-guided therapy. Despite a neutral impact on clinical efficacy, we believe our results represent a substantial benefit to both our patients and institution due to the safety and operational advantages of the dosing and monitoring approach used. All patients in our study were receiving vancomycin for a culture-proven gram-positive infection, highlighting the important role this agent continues to play in the treatment of these infections. It is worth noting that efforts to reduce VA-AKI should also focus on limiting unnecessary use of vancomycin, and the benefits of such a stewardship initiative may exceed those described in our study population as vancomycin is often used empirically.

## CONCLUSIONS

Use of MIPD and single-concentration monitoring improved overall outcomes among patients receiving AUC-guided vancomycin for culture-proven gram-positive infections compared to patients receiving trough-guided therapy. Safety was improved most notably due to reduced incidence of VA-AKI, and no concerns for reduced therapeutic efficacy were observed. In addition to favorable clinical outcomes, MIPD allowed for earlier assessment of AUC target attainment and allowed for greater flexibility in the collection of serum vancomycin concentrations.

## Notes

***Author contributions*.** N. M. H., W. S. E., M. L. B., and J. S. developed the objectives and methods of the study. N. M. H. and W. C. collected the data. R. A. O. conducted data analyses. N. M. H. drafted the initial version of the manuscript and developed the tables and figures. All authors reviewed and approved the final version of the manuscript as submitted.

***Acknowledgments.*** This work was supported by the UAB Antimicrobial Stewardship Program and UAB Pharmacy Department leadership. The authors thank Rachel White, Bryan Cook, Myra Collier, and other participants of the project workgroup for their expertise and assistance implementing vancomycin MIPD at our institution. We thank the UAB pharmacists who use MIPD on a daily basis to ensure provision of safe, effective vancomycin therapy.

***Financial support.*** This study was partially supported by the National Institutes of Health (cooperative agreement number UL1 TR003096 to R. A. O.). ***Patient consent.*** This study was approved by the Institutional Review Board at the University of Alabama at Birmingham with a waiver of informed consent due to the study design.
